# PREDICTORS OF FALLS IN OLDER ADULTS ACROSS PARTNER SETTINGS

**DOI:** 10.1093/geroni/igz038.665

**Published:** 2019-11-08

**Authors:** Helen W Lach, Theodore Malmstrom

**Affiliations:** 1 Saint Louis University, Missouri, United States; 2 Saint Louis University, St. Louis, Missouri, United States

## Abstract

Falls cause significant morbidity and mortality in older adults. We explored predictors of falls in older adults completing Rapid Geriatric Assessments in Missouri (N=7796). Assessments were completed by health professionals and students at community sites (n= 1817), physician practices/clinics (n= 4441), nursing homes (n=525), and hospitals (n=1013). Multinomial regression analyses were used to identify predictors of falls from: age, gender, frailty score, self-reported walking ability, and cognition. Participants had a mean age of 78.4±8.4, were 65.2% female, 41.7% fallers, 28.1% frail, and 40.5% pre-frail. All variables except gender were associated with falls. For the total sample, frailty (OR 2.11 for 1-3 falls, OR 3.74 for 4+ falls; p<.001) and self-reported walking difficulty (OR 1.85 for 1-3 falls, OR 2.36 for 4+ falls; p<.001) had the highest odds ratios and were consistent predictors of falls in site-specific analyses. Self-rated walking, age, and cognition were risk factors in some settings.

